# Supplementary material for: Prevalence and associated factors of active trachoma among 1–9 years of age children in Andabet district, northwest Ethiopia, 2023: A multi-level mixed-effect analysis
Source: PLoS Negl Trop Dis. 2023 Aug 17;17(8):e0011573. doi: 10.1371/journal.pntd.0011573 (PMC10464999; doi:10.1371/journal.pntd.0011573)
Supplement: Attachment — Submitted filename: response for reviewer comments.docx [file pntd.0011573.s003.docx]

**Authors’ response to Editor’s and Reviewer’s comments**

**The title “Prevalence and associated factors of active trachoma among 1-9 years of age children in Andabet district, northwest Ethiopia, 2023: a multi-level mixed-effect analysis”**

Dear Editor, we have revised our manuscript based on the reviewer's suggestions and comments.  Here is a response to the reviewer's and your comments and suggestions.

**Author's response to the editor’s suggestions/comments**

Thank you very much for submitting your manuscript "Prevalence and associated factors of active trachoma among 1-9 years of age children in Andabet district, northwest Ethiopia, 2023: a multi-level mixed-effect analysis" for consideration at PLOS Neglected Tropical Diseases. As with all papers reviewed by the journal, your manuscript was reviewed by members of the editorial board and by several independent reviewers. The reviewers appreciated the attention to an important topic. Based on the reviews, we are likely to accept this manuscript for publication, providing that you modify the manuscript according to the review recommendations.

**Author Response:** thank you very much, dear editor, for your grand and intriguing notion regarding the manuscript, we have revised the manuscript according to the reviewer’s recommendations.

When you are ready to resubmit, please upload the following:

[1] A letter containing a detailed list of your responses to the review comments and a description of the changes you have made in the manuscript. Please note while forming your response, if your article is accepted, you may have the opportunity to make the peer review history publicly available. The record will include editor decision letters (with reviews) and your responses to reviewer comments. If eligible, we will contact you to opt-in or out.

**Author Response:** thank you, dear editor, we have prepared both the letter containing a detailed list of our responses to the reviewer’s comments and a description of the changes we have made in the manuscript.

[2] Two versions of the revised manuscript: one with either highlights or tracked changes denoting where the text has been changed; the other a clean version (uploaded as the manuscript file).

**Author Response:** we have prepared the two versions of the manuscript one with tracked change and the other a clean version

Figure Files:

While revising your submission, please upload your figure files to the Preflight Analysis and Conversion Engine (PACE) digital diagnostic tool, https://pacev2.apexcovantage.com. PACE helps ensure that figures meet PLOS requirements. To use PACE, you must first register as a user. Then, login and navigate to the UPLOAD tab, where you will find detailed instructions on how to use the tool. If you encounter any issues or have any questions when using PACE, please email us at [figures@plos.org](mailto:figures@plos.org).

**Author Response:** thank you, dear editor, we have uploaded the figure with PACE

Data Requirements:

Please note that, as a condition of publication, PLOS' data policy requires that you make available all data used to draw the conclusions outlined in your manuscript. Data must be deposited in an appropriate repository, included within the body of the manuscript, or uploaded as supporting information. This includes all numerical values that were used to generate graphs, histograms etc.. For an example see here: <http://www.plosbiology.org/article/info%3Adoi%2F10.1371%2Fjournal.pbio.1001908#s5>.

**Author Response:** thank you, dear editor**,** the data used for this study was included with the submission as a supporting information-compressed zip file.

References
please review your reference list to ensure that it is complete and correct. If you have cited papers that have been retracted, please include the rationale for doing so in the manuscript text, or remove these references and replace them with relevant current references. Any changes to the reference list should be mentioned in the rebuttal letter that accompanies your revised manuscript. If you need to cite a retracted article, indicate the article's retracted status in the References list and also include a citation and full reference for the retraction notice.

**Author Response:** thank you very much, dear editor, for reminding us to review the reference list. We ensured it’s complete and correct. And also we have not cited any papers that have been retracted.

**Response to the reviewer's comment**

Dear reviewer, we are gratified and admired your heedful and scrupulous reading of this manuscript and we would like to say thank you for your solicitous and valuable comments and suggestions. Based on your comments and suggestions, we had carefully revised the manuscript.

1. Reviewer #1: This is a very improved version of the manuscript that needs only very minor grammatical corrections as listed below

**Author Response:** thank you very much, dear reviewer, Based on your comments and suggestions, we have done the revision.

In the Abstract:

Background:

Fifth line - "was 37%. Then space and capital "W" for With.

Seventh line - TF mentioned in full above in fourth and fifth lines but TI not mentioned in full. Plus ass TI in full with Acronym in seventh line as some people may read only the abstract.

Conclusion:

First line - Write WHO in full then the Acronym as some people may read only abstract.

Fourth line - use "paying" instead of "taking", reads better as Therefore, paying special attention ......."

**Author Response**: thank you very much, dear reviewer, Based on your comments and suggestions, we have done the revision.

3. In the Main body:

Abstract: I suggest same corrections as above in Lines 38, 40, 57 and 59.

Line 65: synchronise with Line 57; write in full with Acronym in bracket in line 57 and then write WHO's in line 65.

**Author Response:** thank you very much, dear reviewer, Based on your comments and suggestions, we have done the revision.

Line 144: I am not familiar with the term "pen spiring". Please cross check and amend if necessary.

**Author Response**: thank you very much, dear reviewer, for reminding us to cross check. We amend the word to "pen spinning" i.e. to randomize the starting location of the village, we carried out pen spinning.

Line 293: Better to use "because " instead of "due to" so the sentence reads well; "This might be because a discharge from the infected eye prompts transmission of infection by direct contact or via fingers, flies, or fomites (38)".

Line 299: Better to use "because " instead of "due to" so the sentence reads well.

Line 300: Use "are" instead of "is" reference is to "eye-questing flies and other domestic Muscidae. Also, use "for" instead of "in" and plural "open" instead of singular "opens as I believe reference here is to the word "vectors". Sentence reads better (Lines 299 and 300) as: This might be because the eye-questing flies Musca sorbents and other domestic Muscidae are vectors for Chlamydia trachomatis and open the trachoma transmission route (39).

Line 305: Please add "that have" to "been" so the sentence (Lines 304 and 305) reads well; The possible reason might be due to Musca sorbents; these are a reservoir of the causative agent, Chlamydia trachomatis, that have been shown to preferentially breed in human excreta (13).

Line 316: Remove the full stop before "There".

**Author Response:** thank you very much, dear reviewer, Based on your comments and suggestions, we have done the revision.
